# Electrochemical Epoxidation Catalyzed by Manganese Salen Complex and Carbonate with Boron-Doped Diamond Electrode

**DOI:** 10.3390/molecules28041797

**Published:** 2023-02-14

**Authors:** Pijush Kanti Roy, Keisuke Amanai, Ryosuke Shimizu, Masahito Kodera, Takuya Kurahashi, Kenji Kitayama, Yutaka Hitomi

**Affiliations:** 1 Department of Molecular Chemistry and Biochemistry, Graduate School of Science and Engineering, Doshisha University, Kyotanabe, Kyoto 610-0321, Japan; 2 Department of Nutrition Science, University of Nagasaki, Siebold Campus 1-1-1 Manabino, Nagayo, Nishi-Sonogi, Nagasaki 851-2195, Japan; 3 PRESTO/JST, 4-1-8 Honcho, Kawaguchi, Saitama 332-0012, Japan; 4 R & D Material SBU, Daicel Corporation, Ofuka-cho, Kita-ku, Osaka 530-0011, Japan

**Keywords:** boron-doped diamond, electrochemical epoxidation, manganese salen complex, percarbonate

## Abstract

Epoxides are essential precursors for epoxy resins and other chemical products. In this study, we investigated whether electrochemically oxidizing carbonate ions could produce percarbonate to promote an epoxidation reaction in the presence of appropriate metal catalysts, although Tanaka and co-workers had already completed a separate study in which the electrochemical oxidation of chloride ions was used to produce hypochlorite ions for electrochemical epoxidation. We found that epoxides could be obtained from styrene derivatives in the presence of metal complexes, including manganese(III) and oxidovanadium(IV) porphyrin complexes and manganese salen complexes, using a boron-doped diamond as the anode. After considering various complexes as potential catalysts, we found that manganese salen complexes showed better performance in terms of epoxide yield. Furthermore, the substituent effect of the manganese salen complex was also investigated, and it was found that the highest epoxide yields were obtained when Jacobsen’s catalyst was used. Although there is still room for improving the yields, this study has shown that the in situ electrochemical generation of percarbonate ions is a promising method for the electrochemical epoxidation of alkenes.

## 1. Introduction

**Figure 1:**
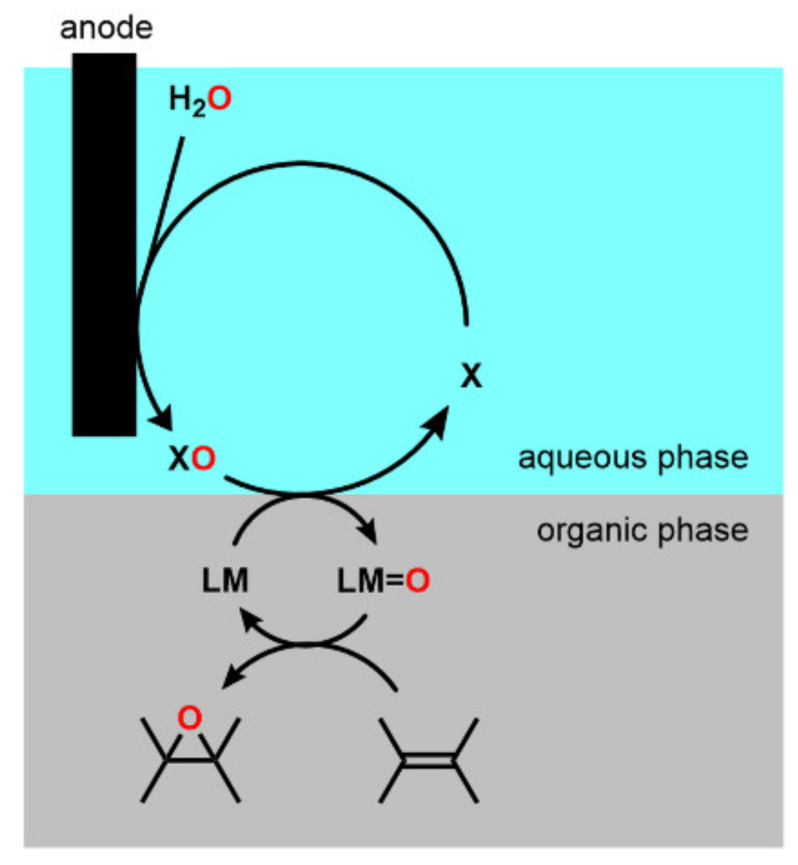
Epoxidation reaction with the electrochemical oxidative generation of an oxidizing agent (XO).

**Figure 2:**
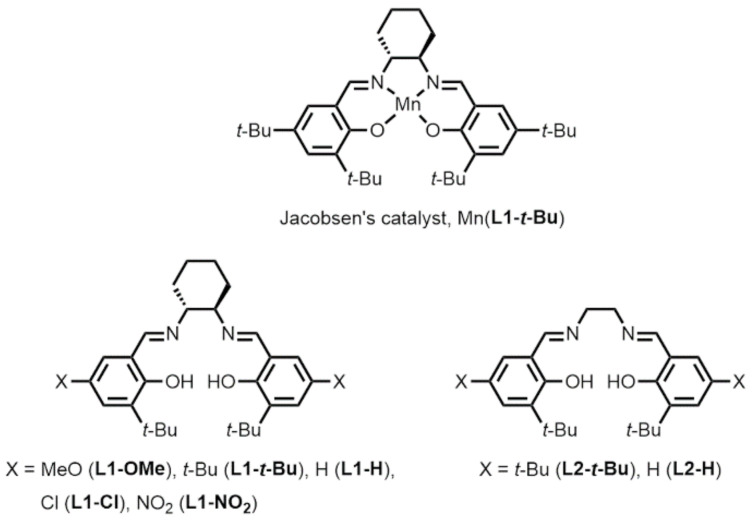
Jacobsen’s catalyst and salen ligands (and their abbreviations).

**Figure 3:**
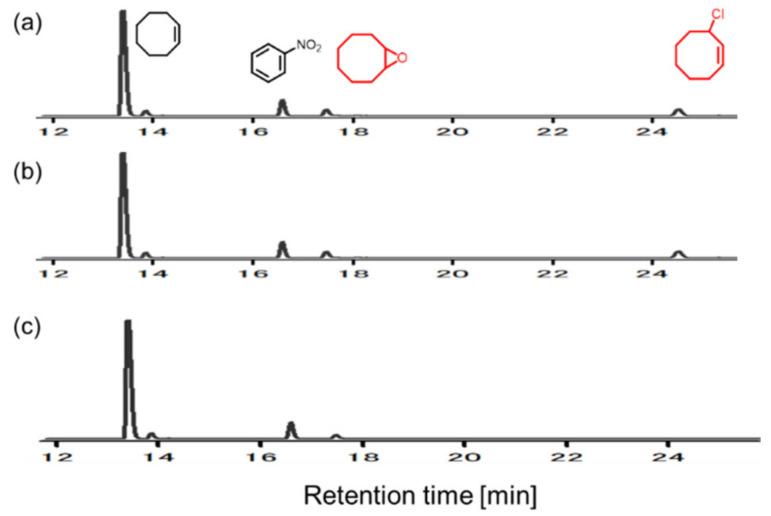
Gas chromatograms for electrochemical cyclooctene epoxidation catalyzed by Mn(L1-*t*-Bu) under different reaction conditions. (**a**) CH_2_Cl_2_/1 M NaCl aq. (1:9), (+)Pt/(−)Pt, (**b**) CH_2_Cl_2_/1 M NaCl aq. (1:9), (+)BDD/(−)Pt, and (**c**) CH_2_Cl_2_/1 M Na_2_CO_3_ aq. (1:9), (+)BDD/(−)Pt. Other reaction conditions: substrate (0.25 mmol), catalyst (0.05 mmol), 2.5 V vs. Ag/AgCl, in undivided cell, 0 °C (bath temp.), 90 min.

Epoxide functional groups are essential not only in the chemical and pharmaceutical industries but also in nature. In the chemical and pharmaceutical industries, epoxide-containing compounds are used as versatile intermediates in the manufacture of many chemical products, including epoxy resins, fragrances, and pharmaceuticals. In nature, many natural products have epoxide groups and are used for medical purposes or as essential compounds for organic synthesis because they are highly reactive. Epoxide can be synthesized using different reactions, including the chlorohydrin method, but is often prepared through the transfer of oxygen atoms to alkenes using peroxides such as *tert*-butyl hydroperoxide, peracetic acid, and *m*-chloroperoxybenzoic acid [1,2,3]. Such reagents are difficult to handle and produce undesirable stoichiometric by-products, except for hydrogen peroxide [4,5]. These problems can be avoided if the compounds that are by-products of the reaction between the alkene and the oxygen additive can be oxidized and the oxygen additive can be synthesized again [6,7,8,9,10,11,12,13]. For example, the two-electron reduction of oxygen can produce hydrogen peroxide. It should be noted that Collman and co-workers pioneered the epoxidation of alkene using manganese *meso*-tetraphenylporphyrin catalysts with hydrogen peroxide generated through the electrochemical reduction of oxygen using polymer-coated electrodes [11]. Electrocatalytic epoxidation using manganese salen complexes also has been performed through reductive dioxygen activation [14,15]. It is also possible to synthesize hydrogen peroxide, alky or acyl peracids, or oxygen-transfer reagents by electrode oxidation using water as an oxygen source, as shown in Figure 1. Recently, Manthiram and co-workers reported a new electrochemical method for the epoxidation of an alkene substrate using water as the sole source of oxygen atoms and monodisperse manganese oxide nanoparticles as catalysts [6]. Based on electro-kinetic studies, they proposed the formation of manganese(IV) oxo species as the resting state as well as the oxidizing species for alkene epoxidation. In addition, photochemical epoxidation using various metal complexes with water as the oxygen source have been investigated [8,9,10,16]. For example, chemical oxidants such as potassium hexachloroplatinate and visible light irradiation activate ruthenium(II) porphyrins have been used to form ruthenium oxo species using water as the oxygen atom source, which can transfer oxygen to alkene substrates to form epoxides. Similarly, the photosensitizer tris(2,2′-bipyridyl)ruthenium(II) chloride in combination with the one-electron oxidant pentaamminechlorocobalt(III) chloride produces metal–oxygen species that have been used as catalysts for alkene epoxidation. [(bTAML)Fe^III^-OH_2_]^−^ (bTAML = biuret-modified tetraamido macrocyclic ligand), [(*R*,*R*-BQCN)Mn^II^]^2+^ (*R*,*R*-BQCN = *N*,*N’*-dimethyl-*N*,*N*-bis(8- quinolyl)cyclohexanediamine), and [Ru (TMP)(CO)] (TMP = tetramesitylporphyrin) catalysts have shown high selectivity and yield in photochemical epoxidation. Torii and co-workers produced electrochemical alkene epoxidation reactions using the acetonitrile–water–sodium bromide water–organic solvent biphasic system [12]. In their system, the electrolysis of the bromide ions in the water produces hypobromite ions, which transfer an oxygen atom to the alkene substrates, and the water is an oxygen source for epoxide formation. Tanaka and co-workers subsequently carried out the electrochemical epoxidation of styrene derivatives using *N*,*N*’-bis(3,5-di-*tert*-butylsalicylidene)-1,2-cyclohexanediaminomanganese(III) chloride (Figure 2, Mn(L1-*t*-Bu), known as Jacobsen’s catalyst) in a dichloromethane–water–sodium chloride water–organic solvent biphasic system [17]. In this new system, the electrolysis of the chloride ions in the water produces hypochlorite ions, and Jacobsen’s catalyst promotes the epoxidation reaction of the styrene derivatives with the hypochlorite ions in a water–organic solvent biphasic system. Thus, Jacobsen’s catalyst may produce manganese-oxo species which can serve as the active species for alkene epoxidation [18]. These excellent results demonstrate that water is an attractive and safe source of oxygen atoms in electrochemical and photochemical epoxidation reactions. With this in mind, we re-investigated and checked the scope and limitations of the method for the electrochemical epoxidation of alkenes reported by Tanaka and co-workers [17]. Firstly, cyclooctene was electrochemically epoxidized in the dichloromethane and aqueous sodium chloride solution system using the same catalyst, Jacobsen’s catalyst Mn(L1-*t*-Bu), according to the procedure reported by Tanaka and co-workers [17]. As a result, we found that, besides the desired epoxide, an undesired product that chlorinated at the allylic position of the cyclooctene was generated (Figure 3). It is known that the electrochemical oxidation of chloride ions in water produces active chlorine species such as Cl_2_ and Cl_2_O [19] which may cause chlorination at the allylic position of cyclooctene. We therefore searched for a suitable chemical species to replace the chloride ions in electrochemical epoxidation reactions in order to avoid the allylic halogenation of alkenes.

Carbonates have often been used as activators for oxidation reactions with hydrogen peroxide, including epoxidation reactions [20,21,22,23,24]. It has been reported that percarbonate is involved in manganese-catalyzed alkene epoxidation reactions using hydrogen peroxide in carbonate or bicarbonate aqueous solutions [20,21,24,25,26]. Burgess and co-workers proposed the involvement of manganese *η*^2^-peroxycarbonate complexes to facilitate the cleavage of the O–O bonds of percarbonate ions. A similar iron(III) *η*^2^-percarbonate complex was isolated and characterized using X-ray crystallography [27]. Percarbonate is generated through the equilibrium reaction between carbonate ions and hydrogen peroxide [28]. Therefore, the formation of percarbonate is the same as the formation of hydrogen peroxide. It has been reported that percarbonate can be generated via the oxidative electrolysis of carbonate ions using a platinum anode [29,30,31,32,33,34,35,36,37,38] or a boron-doped diamond (BDD) [32,33,34,35,36,37,38] electrode. Compared with other electrodes, a BDD has a higher overpotential for water oxidation [39,40], which is suitable for the electro-synthesis of oxidants in aqueous media. Recently, Wenderich, Mei, and co-workers reported that BDDs are promising tools for anodic hydrogen peroxide production, which is greatly improved by using sodium carbonate as an electrolyte [41]. In this study, we aimed to utilize the electrochemically generated percarbonate ions on a BDD electrode.

Electrocatalysis employing metal complexes has attracted a great deal of attention in the field of organic synthesis and has shown significant progress in recent years [42,43,44,45]. In this study, inspired by Tanaka’s work [17], we have examined electrochemical epoxidation via in situ electrochemical generation of percarbonate by using various metal complexes, including manganese salen complexes and manganese and vanadium porphyrin complexes as a mediator in an organic solvent–aqueous carbonate two-phase system in a simple undivided cell. As we expected, the electrochemical epoxidation of styrene derivatives proceeded when a BDD was used as an anode. The reaction optimization was performed in detail.

## 2. Results and Discussion

### 2.1. Electrochemical Epoxidation Using a BDD

First, to examine whether percarbonate-mediated electrochemical epoxidation can proceed using a BDD, glassy carbon, graphite, or platinum anode, we performed the electrochemical epoxidation of *trans*-β-methylstyrene in a biphasic system containing 1 M sodium carbonate aqueous solution and a solution of Jacobsen’s catalyst Mn(L1-*t*-Bu) in dichloromethane under conditions similar to those used in Tanaka’s work [17]. After 30 min of electrolysis using a BDD as an anode with a voltage of 2.50 V vs. Ag/AgCl at a bath temperature of −5 °C, the epoxide of the *trans*-β-methylstyrene was obtained with a yield of 18.5%, while the electrolysis experiments conducted using the other electrodes (glassy carbon, graphite, or platinum anodes) gave yields lower than 2.2% (Table 1). Thus, the BDD anode showed the best performance in the electrochemical epoxidation of the *trans*-β-methylstyrene. It is thought that the oxidation of water to oxygen is easier with electrodes other than BDDs [39,46]. However, yields decreased with longer reaction times using BDDs. Interestingly, the same product yields were observed under air and nitrogen. When sodium sulfate and sodium chloride were used as an electrolyte instead of sodium carbonate, the yields of the corresponding epoxide of the *trans*-β-methylstyrene were lower than 6%.

Next, we investigated the effects of applied oxidation voltage vs. Ag/AgCl, bath temperature, and type of electrolyte on electrochemical epoxidation. The applied oxidation voltage was very sensitive to the epoxidation reaction of the *trans*-β-methylstyrene. The yields of the corresponding epoxide were 5.7%, 18.5%, and 6.2% for the electrolysis using a BDD electrode at 2.25 V, 2.50 V, and 2.75 V vs. Ag/AgCl, respectively. These results suggest that the production of percarbonate requires more than 2.50 V vs. Ag/AgCl, but that the higher applied voltage may result in unwanted side reactions. The reactions were performed at three different bath temperatures. The yields of the corresponding epoxide from the *trans*-β-methylstyrene were 9.6, 17.5, and 18.5 at bath temperatures of 5 °C, 0 °C, and −5 °C, respectively. Thus, the lower bath temperatures afforded higher epoxide yields, probably due to the suppression of unwanted side reactions at higher temperatures. We also conducted the electrochemical epoxidation of *trans*-β-methylstyrene in different water–organic solvent systems (i.e., water–ethyl acetate, water–acetonitrile, and water–toluene), where the water phase was 1 M sodium carbonate and a BDD electrode was used as an anode with a voltage of 2.50 V vs. Ag/AgCl at a bath temperature of −5 °C. The epoxide yields from the *trans*-β-methylstyrene were lower than 1.3% when ethyl acetate, acetonitrile, and toluene were used, yields which are lower than that obtained in the water–dichloromethane biphasic system (18.5%). In the water–dichloromethane biphasic system, the BDD anode contacts the upper aqueous layer, not the lower dichloromethane phase containing Jacobsen’s catalyst Mn(L1-*t*-Bu) and the *trans*-β-methylstyrene. This alignment allows the efficient production of percarbonate in the aqueous phase and avoids the direct oxidation of the *trans*-β-methylstyrene, which should be one of the key factors in promoting the electrochemical epoxidation reaction in the water–dichloromethane biphasic system.

Next, we examined the electrochemical epoxidation of *cis*-β-methylstyrene, cyclooctene, *trans*-stilbene, and cyclohexene under the optimized conditions using a BDD anode. The epoxide yields are listed in Table 2. We compared the obtained yields with their corresponding alkene ionization because a clear correlation between the alkene ionization potential and the epoxidation rate constants for the iron(IV)-oxo porphyrin cation radical complexes has been reported [47]. As expected, the results show that as the ionization energy of the alkenes increased, the epoxide yield decreased, except in the case of the *trans*-stilbene. The exceptionally low yield we observed may be attributable to the two bulky phenyl groups of the *trans*-stilbene. The correlation between the epoxide yields and the alkene ionization potentials suggests that the epoxidation step should take longer than the formation of percarbonate ions through electrolysis on the BDD anode. As control experiments, we performed epoxidation reactions of the alkenes without using the BDD electrode by adding one equivalent of hydrogen peroxide as an oxidant to a biphasic system containing 1 M sodium carbonate aqueous solution and a solution of Jacobsen’s catalyst Mn(L1-*t*-Bu) in dichloromethane. The epoxide yields obtained via the conventional epoxidation reaction using hydrogen peroxide with sodium carbonate are listed in the right column in Table 1. The epoxide yields obtained using the hydrogen peroxide showed a similar trend to those observed with electrochemical epoxidation. The results suggest that hydrogen peroxide is generated through electrolysis using a BDD anode. In carbonate aqueous solutions, there should be an equilibrium between hydrogen peroxide and percarbonate. Therefore, it is not easy to distinguish between the generation of hydrogen peroxide and the generation of percarbonate. However, it is likely that percarbonate is first generated through the oxidation of carbonate, as reported by Richardson and co-workers, based on their kinetic study using ^13^C-labeled bicarbonate solutions [48].

When *cis*-β-methylstyrene is used as a substrate for an epoxidation reaction, *cis*-to-*trans* isomerization occurs [49,50]. Tanaka and co-workers reported that when the electrochemical epoxidation of *cis*-β-methylstyrene is catalyzed by Jacobsen’s catalyst Mn(L1-*t*-Bu) in the dichloromethane and sodium chloride aqueous solution biphasic system, a corresponding epoxide was obtained with a *cis* to *trans* ratio of 87:13. In our case, under the electrochemical epoxidation conditions, the *cis* to *trans* ratio of the corresponding epoxide obtained from the *cis*-β-methylstyrene was 85:15. A similar *cis* to *trans* isomerization value was observed for the conventional epoxidation reaction of *cis*-β-methylstyrene catalyzed by Jacobsen’s catalyst Mn(L1-*t*-Bu) using hydrogen peroxide. A comparable extent of *cis*-to-*trans* isomerization from *cis*-β-methylstyrene has been observed following an epoxidation reaction with sodium hypochlorite and Jacobsen’s catalyst Mn(L1-*t*-Bu) [49,50]. Electrochemical epoxidation using an optically active (*R*,*R*)-Jacobsen’s catalyst Mn(L1-*t*-Bu) afforded the desired *cis*-(1*R*,2*S*)-epoxide with an enantiomeric excess (ee) of 87%, which was comparable to the reported ee values [17]. The results suggest that the same oxidizing species derived from Jacobsen’s catalyst Mn(L1-*t*-Bu) are formed in the cases of electrochemical epoxidation using chloride ions and carbonate ions as well as conventional epoxidation using hydrogen peroxide with carbonate ions and sodium hypochlorite.

### 2.2. Exploration of More Efficient Catalysts

Next, we examined whether other catalysts could be used for electrochemical epoxidation. In this study, we focused on catalysts that have been reported to be effective in the epoxidation of alkenes with hydrogen peroxide. Lane and Burgess have reported that manganese sulfate catalyzes the epoxidation of alkene using aqueous hydrogen peroxide in the presence of sodium bicarbonate [51]. They assumed that percarbonate ions are formed from hydrogen peroxide and that bicarbonate ions react in situ with manganese ions to give the active intermediate for the epoxidation reactions of alkenes. Qi and co-workers have reported that manganese oxide has superior properties for converting styrene to epoxide with aqueous hydrogen peroxide in a bicarbonate solution [52]. Based on these reports, we conducted the electrochemical epoxidation of *trans*-β-methylstyrene under the optimal conditions with a BDD electrode as an anode with a voltage of 2.50 V vs. Ag/AgCl at a bath temperature of −5 °C, using manganese sulfate or manganese oxide as a catalyst. However, these two reactions using manganese sulfate or manganese oxide instead of Jacobsen’s catalyst Mn(L1-*t*-Bu) afforded negligible amounts of epoxide from the *trans*-β-methylstyrene. We further examined whether conventional epoxidation using hydrogen peroxide proceeds in the 1 M sodium carbonate–dichloromethane biphasic system containing manganese sulfate or manganese oxide as a catalyst. However, the yields of the corresponding epoxide were negligible. These results indicate that our biphasic system is not suitable for epoxidation reactions using manganese sulfate or manganese oxide as a catalyst, probably because these catalysts are water soluble or hydrophilic.

Dar and co-workers have reported that the electron-deficient oxidovanadium(IV) porphyrin catalyst β-octabromo-meso-tetrakis(2,6-dibromo-3,5-dimethoxyphenyl)porphyrinatooxidovanadium (VO(L1)) efficiently promotes the epoxidation of styrene using hydrogen peroxide in acetonitrile–water containing sodium bicarbonate [53]. Nishihara and co-workers have carried out electrochemical cyclooctene epoxidation with manganese(III) porphyrin complexes using hydrogen peroxide generated from polymer-coated electrodes in dichloromethane containing 1-methylimidazole and benzoic acid [11].

Thus, some oxidovanadium(IV) porphyrin and manganese(III) complexes promote the chemical epoxidation of alkenes with hydrogen peroxide in the presence of bicarbonate or electrochemical epoxidation. Therefore, we examined the electrochemical epoxidation of *trans*-β-methylstyrene under the optimal conditions for Jacobsen’s catalyst Mn(L1-t-Bu), i.e., using a BDD electrode as an anode with a voltage of 2.50 V vs. Ag/AgCl at a bath temperature of −5 °C, with manganese(III) and oxidovanadium(IV) tetraphenylporphyrin derivatives as a catalyst. We prepared six metalloporphyrins for this purpose in accordance with the literature [53]: manganese tetraphenyl porphyrin, Mn(TPP), manganese 5,10,15,20-tetrakis(2,6-dichlorophenyl)porphyrin, Mn(TDCPP), 5,10,15,20-tetrakis(3,5-dimethoxyphenyl)porphyrin, Mn(L2), oxidovanadium(IV) tetraphenylporphyrin, VO(TPP), oxidovanadium(IV) 5,10,15,20-tetrakis(2,6-dibromo-3,5-dimethoxyphenyl)porphyrin VO(L1), and oxidovanadium(IV) 5,10,15,20-tetrakis(3,5-dimethoxyphenyl)porphyrin, VO(L2). The chemical structures of the catalysts are shown in Figure 2 together with the epoxide yields obtained from the *trans*-β-methylstyrene.

Among the oxidovanadium(IV) porphyrins, electron-deficient oxidovanadium(IV) porphyrin VOL1 showed the lowest epoxide yield derived from the *trans*-β-methylstyrene (0.6%), probably because of its low solubility in 1 M sodium carbonate–dichloromethane biphasic solution. VOTPP and VOL2 showed respective epoxide yields of 9.6% and 5.3% derived from the *trans*-β-methylstyrene. Among the manganese porphyrin complexes, MnTPP produced the highest yield (6.2%). Mn(TDCPP) is known as a more robust oxidation catalyst than Mn(TPP). Regardless, we found that the use of MnL2 as a catalyst produced only negligible amounts of epoxide, probably due to its lower solubility in 1 M sodium carbonate–dichloromethane biphasic solution. Oxidovanadium(IV) porphyrins are neutral compounds, but manganese(III) porphyrins are cationic. This difference may affect their solubility in the biphasic solution. For example, VO(L2) showed a higher yield (9.6%) compared with its manganese counterpart, MnL2 (0.8%). Thus, our catalyst survey disclosed that Jacobsen’s catalyst Mn(L1-*t*-Bu), a manganese salen complex, is superior to oxidovanadium(IV) and manganese porphyrins.

### 2.3. Optimization of Manganese Salen Complexes

**Figure 4:**
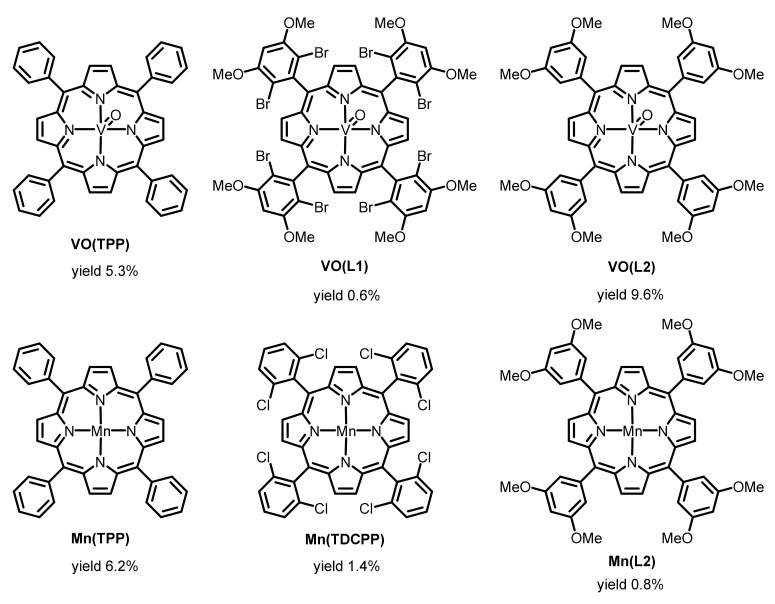
Oxidovanadium(IV) porphyrin and manganese porphyrin catalysts are shown with the yields of epoxide obtained from the *trans*-β-methylstyrene under the electrochemical conditions.

Next, we decided to optimize the structures of the manganese salen complexes for the electrochemical epoxidation reaction we established in this study. For this purpose, we prepared eight manganese complexes, including Jacobsen’s catalyst Mn(L1-*t*-Bu) [54]. The manganese salen complexes had *trans*-1,2-cyclohexanediamine or 1,2-ethylenediamine as a backbone, and a methoxy-, chloride-, nitro-, and *tert*-butyl group at the 4-position of the phenol moieties, whose chemical structures are shown in Figure 4. The epoxide yields derived from *trans*-β-methylstyrene after a 30 min reaction under the optimal electrochemical epoxidation conditions are listed in Table 3. For comparison, we also performed conventional chemical epoxidation using hydrogen peroxide as an oxidant in the same 1 M sodium carbonate and dichloromethane biphasic system. A similar trend in the substituent effect was observed for all the manganese complexes in both the electrochemical and conventional epoxidation reactions. Among them, Jacobsen’s catalyst Mn(L1-*t*-Bu) showed the highest yield for both types of epoxidation reaction. The redox potentials of the manganese complexes had been evaluated by one of our authors and were reported to be in the order: Mn(L1-Ome) < Mn(L1-*t*-Bu) < Mn(L1-Cl) [54]. In terms of the redox potentials, the manganese-oxo species of Mn(L1-Cl) should have been more reactive for the epoxidation than Mn(L1-*t*-Bu), but the epoxide yield observed with Mn(L1-Cl) was much lower than that of Mn(L1-*t*-Bu). Thus, the reactivity of the oxidizing species of manganese salen complexes cannot explain the epoxide yields. This opposite trend could be attributable to the formation step of the oxidizing species of manganese salen complexes. We speculated that Mn(L1-Cl) could not react with percarbonate to form the corresponding manganese-oxo species due to the electron-withdrawing groups on the phenyl groups. Therefore, according to the established procedure, we performed the epoxidation reaction using a more potent oxidizing agent, sodium hypochlorite, only in dichloromethane [18]. The data are listed in the right column of Table 3. Jacobsen’s catalyst Mn(L1-*t*-Bu) gave the highest yield, a result observed for the electrochemical epoxidation with carbonate as well as for the conventional epoxidation using the hydrogen peroxide with carbonate system. However, the epoxidation reaction using the other manganese salen complexes and sodium hypochlorite only in dichloromethane and sodium hypochlorite showed higher epoxide yields than the reactions in the 1 M sodium carbonate and dichloromethane biphasic system. These results demonstrate the uniqueness of Jacobsen’s catalyst Mn(L1-*t*-Bu) as a useful and practical epoxidation catalyst. We speculate that its electronic structure, bulky functional groups, rigid backbone, and high solubility in organic solvents all contribute to its catalytic capability.

Next, we examined epoxidation reactions involving cyclooctene using the above manganese salen complexes. The cyclooctene epoxide yields obtained by electrochemical epoxidation are listed in Table 4, together with the cyclooctene epoxide yields obtained using hydrogen peroxide. Cyclooctene is a less reactive substrate than trans-β-methylstyrene. The cyclooctene epoxide yields were 5.6% and 6.8% after 30 min and 90 min, respectively (Table 3 and Table 4), which are lower than that observed with trans-β-methylstyrene (18.5%) after 30 min. A similar trend was observed in the epoxidation yield of cyclooctene as that observed with *trans*-β-methylstyrene. Jacobsen’s catalyst Mn(L1-*t*-Bu) gave the highest yield in both electrochemical epoxidation and epoxidation with hydrogen peroxide with carbonate. Overall, it is clear that the epoxide yields are not dependent simply on the electronic properties of Mn salen complexes. These results again illustrate that Jacobsen’s catalyst Mn(L1-*t*-Bu) is an excellent electrochemical epoxidation catalyst. We tentatively concluded that the bulkiest catalyst, Jacobsen’s catalyst Mn(L1-*t*-Bu), favors the formation of the active mononuclear manganese-oxo species because four *tert*-butyl groups on Jacobsen’s catalyst prevent the formation of inactive *μ*-oxo dimers, resulting in a higher effective concentration of the active catalyst in solution compared with other less bulky Mn(salen) complexes [55]. The lower yields observed with Mn(L2-*t*-Bu) and Mn(L2-H) also suggest the substantial role of the cyclohexane moiety of L1 in the epoxidation reaction.

## 3. Experimental

### 3.1. Chemicals and Equipment

Cyclohexene and cyclooctene were passed through a short alumina column prior to their use. Except for Mn(L1-*t*-Bu), the Mn(salen) complexes were prepared according to the reported procedure [54]. All other chemicals and solvents, including the Mn(L1-*t*-Bu), were purchased and used without further purification. The oxidation products of *trans*-stilbene were analyzed using ^1^H NMR measurements obtained with a JEOL JMN-A500 NMR spectrometer. The other products were analyzed using Shimadzu GC-2014 and GCMS-QP2020 NX spectrometers equipped with a GL Science Inert Cap 1701 capillary column (60 m × 0.25 mm).

### 3.2. General Procedure for Electrochemical Epoxidation

Electrochemical epoxidation was performed using IKA ElectraSyn2.0 in a 5 mL vial equipped with a BDD plate as an anode and Pt as a cathode. A mixture of olefin (0.25 mmol), metal complex (0.05 mmol), dichloromethane (0.5 mL), and 1 M Na_2_CO_3_ (4.5 mL) was placed in the 5 mL vial. The vessel was immersed in an alcohol bath, whose temperature was kept at −5 °C with a cooling system (Techno Sigma, Okayama, Japan, UCR-150N-S). The mixture was electrolyzed for the indicated reaction time at a constant voltage, typically 2.5 V vs. Ag/AgCl (3 M NaCl), at a low temperature under air, and with gentle stirring so that the two phases were retained. After the reaction, *p*-nitrotoluene or nitrobenzene (0.1 mmol) was added to the resulting mixture as an internal standard. The product yields and cis/trans ratios were determined using GC analysis of authentic samples. The ee% was determined using HPLC.

### 3.3. General Procedure for Epoxidation with Hydrogen Peroxide

A mixture of olefin (0.25 mmol), metal complex (0.05 mmol), dichloromethane (0.5 mL), and 1 M Na_2_CO_3_ (4.5 mL) was placed in the 5 mL vial. The bath temperature was kept at −2 °C with a cooling system (Techno Sigma, UCR-150N-S). Hydrogen peroxide (0.25 mmol) was then added to the mixture. After the reaction, *p*-nitrotoluene (0.1 mmol) was added to the resulting mixture as an internal standard.

## 4. Conclusions

In conclusion, we confirmed that electrochemical alkene epoxidation proceeds using a BDD electrode, Jacobsen’s catalyst, and carbonate as a mediator in a 1 M sodium carbonate and dichloromethane biphasic system. It should be noted that unwanted chlorination at the allylic position of the alkene does not occur in our method. Epoxide yields were higher than those obtained from electrochemical epoxidation with chloride ions as the mediator when a BDD was used as an anode. We believe that carbonate ions are better mediators for electrochemical epoxidation than chloride ions. Cyclooctene epoxide yields were still low even after prolonged reactions under optimal conditions. The authors are currently attempting to improve the reaction conditions for carbonate-mediated electrochemical epoxidation.

## 5. Patents

The authors have filed a patent application (WO/2021/085268) on the basis of this work.

## Figures and Tables

**Table 1:** Epoxidation of *trans*-β-methylstyrene catalyzed by Mn^III^(L1-*t*-Bu) ^1^.

Anode (3 cm^2^)	Epoxide Yield (%)
BDD	18.5
glassy carbon	2.2
platinum	1.8
graphite	1.8

^1^ Reaction conditions: substrate (0.25 mmol), catalyst (0.05 mmol), CH_2_Cl_2_/1 M Na_2_CO_3_ aq. (1:9), (−)Pt, 2.5 V vs. Ag/AgCl, in undivided cell, −5 °C (bath temp.), 30 min, under air.

**Table 2:** Mn(L1-*t*-Bu)-catalyzed epoxidation of a series of alkenes ^1^.

Substrate (Ionization Energy)	Epoxide Yield (%)	
	Electrochemical Generation	H_2_O_2_/Na_2_CO_3_ ^2^
*trans*-β-methylstyrene	18.5	26.4
(8.08 eV)		
*cis*-β-methylstyrene	19.8 (85:15) ^3^	22.3 (84:16) ^3^
(8.48 eV)		
cyclooctene	5.6	1.4
(9.02 eV)		
*trans*-stilbene	1.6	1.0
(7.70 eV)		
cyclohexene	0.3	0.4
(9.12 eV)		

^1^ Reaction conditions: substrate (0.25 mmol), catalyst (0.05 mmol), CH_2_Cl_2_/1 M Na_2_CO_3_ aq. (1:9), (+)BDD/(−)Pt, 2.5 V vs. Ag/AgCl, in undivided cell, −5 °C (bath temp.), 30 min. ^2^ H_2_O_2_ (0.25 mmol), CH_2_Cl_2_/1 M Na_2_CO_3_ aq. (1:9), −2 °C (bath temp.). ^3^ *trans*/*cis.*

**Table 3:** Mn(L1-*t*-Bu)-catalyzed epoxidation of *trans*-β-methylstyrene ^1^.

Catalyst	Epoxide Yield (%)		
	Electrochemical Generation	H_2_O_2_/Na_2_CO_3_ ^2^	NaOCl ^3^
Mn(L1-Ome)	5.3	2.1	45.5
Mn(L1-*t*-Bu)	18.5	26.4	74.1
Mn(L1-H)	2.2	13.2	59.8
Mn(L1-Cl)	1.8	3.3	60.9
Mn(L1-NO_2_)	1.8	1.8	53.8
Mn(L2-t-Bu)	7.3	1.5	–
Mn(L2-H)	0.5	0.8	–
none	0.7	0.5	8.4

^1^ Reaction conditions: substrate (0.25 mmol), catalyst (0.05 mmol), CH_2_Cl_2_/1 M Na_2_CO_3_ aq. (1:9), (+)BDD/(−)Pt, 2.5 V vs. Ag/AgCl, in undivided cell, −5°C (bath temp.), 30 min. ^2^ H_2_O_2_ (0.25 mmol), CH_2_Cl_2_/1 M Na_2_CO_3_ aq. (1:9), −2 °C (bath temp.). ^3^ Reaction conditions: substrate (0.2 mmol), catalyst (0.01 mmol), NaOCl (0.72 mmol), CH_2_Cl_2_/H_2_O (1:2), 0 °C (bath temp.), 16 h.

**Table 4:** Cyclooctene epoxidation catalyzed by manganese salen complexes ^1^.

Catalyst	Epoxide Yield (%)	
	Electrochemical Generation	H_2_O_2_/Na_2_CO_3_ ^2^
Mn(L1-Ome)	6.8	8.6
Mn(L1-*t*-Bu)	9.9	9.3
Mn(L1-H)	7.7	8.1
Mn(L1-Cl)	8.0	7.6
Mn(L1-NO_2_)	8.1	6.3

^1^ Reaction conditions: substrate (0.25 mmol), catalyst (0.05 mmol), CH_2_Cl_2_/1 M Na_2_CO_3_ aq. (1:9), (+)BDD/(−)Pt, 2.5 V vs. Ag/AgCl, in undivided cell, 0 °C (bath temp.), 90 min. ^2^ H_2_O_2_ (0.25 mmol), CH_2_Cl_2_/1 M Na_2_CO_3_ aq. (1:9), 30 min.

## Data Availability

The data presented in this study are available on request from the corresponding author.

## Sample Availability

Samples of the compounds are not available from the authors.

## References

[1] Yan M., Kawamata Y., Baran P.S. (2017). Synthetic Organic Electrochemical Methods since 2000: On the Verge of a Renaissance. Chem. Rev..

[2] Xia Q.H., Ge H.Q., Ye C.P., Liu Z.M., Su K.X. (2005). Advances in homogeneous and heterogeneous catalytic asymmetric epoxidation. Chem. Rev..

[3] Holm R.H. (1987). Metal-Centered Oxygen Atom Transfer Reactions. Chem. Rev..

[4] Kim C., Traylor T.G., Perrin C.L. (1998). MCPBA epoxidation of alkenes: Reinvestigation of correlation between rate and ionization potential. J. Am. Chem. Soc..

[5] Dryuk V.G. (1976). The mechanism of epoxidation of olefins by peracids. Tetrahedron.

[6] Jin K., Maalouf J.H., Lazouski N., Corbin N., Yang D., Manthiram K. (2019). Epoxidation of Cyclooctene Using Water as the Oxygen Atom Source at Manganese Oxide Electrocatalysts. J. Am. Chem. Soc..

[7] Tatsumi D., Tsukamoto T., Honna R., Hoshino S., Shimada T., Takagi S. (2017). Highly selective photochemical epoxidation of cyclohexene sensitized by Ru(II) porphyrinclay hybrid catalyst. Chem. Lett..

[8] Chandra B., Singh K.K., Gupta S.S. (2017). Selective photocatalytic hydroxylation and epoxidation reactions by an iron complex using water as the oxygen source. Chem. Sci..

[9] Shen D., Saracini C., Lee Y.M., Sun W., Fukuzumi S., Nam W. (2016). Photocatalytic Asymmetric Epoxidation of Terminal Olefins Using Water as an Oxygen Source in the Presence of a Mononuclear Non-Heme Chiral Manganese Complex. J. Am. Chem. Soc..

[10] Funyu S., Isobe T., Takagi S., Tryk D.A., Inoue H. (2003). Highly efficient and selective epoxidation of alkenes by photochemical oxygenation sensitized by a ruthenium(II) porphyrin with water as both electron and oxygen donor. J. Am. Chem. Soc..

[11] Nishihara H., Pressprich K., Murray R.W., Collman J.P. (1990). Electrochemical Olefin Epoxidation with Manganese meso-Tetraphenylporphyrin Catalyst and Hydrogen Peroxide Generation at Polymer-Coated Electrodes. Inorg. Chem..

[12] Torii S., Uneyama K., Tanaka H., Yamanaka T., Yasuda T., Ono M., Kohmoto Y. (1981). Efficient Conversion of Olefins into Epoxides, Bromohydrins, and Dibromides with Sodium Bromide in Water-Organic Solvent Electrolysis Systems. J. Org. Chem..

[13] Torii S., Uneyama K., Ono M., Tazawa H., Matsunami S. (1979). A regioselective ω-epoxidation of polyisoprenoids by the sodium bromide promoted electrochemical oxidation. Tetrahedron Lett..

[14] Moutet J.-C., Ourari A. (1997). Electrocatalytic epoxidation and oxidation with dioxygen using manganese(III) Schiff-base complexes. Electrochim. Acta.

[15] Guo P., Wong K.-Y. (1999). Enantioselective electrocatalytic epoxidation of olefins by chiral manganese Schiff-base complexes. Electrochem. Commun..

[16] Ishikawa A., Sakaki S. (2011). Theoretical study of photoinduced epoxidation of olefins catalyzed by ruthenium porphyrin. J. Phys. Chem. A.

[17] Tanaka H., Kuroboshi M., Takeda H., Kanda H., Torii S. (2001). Electrochemical asymmetric epoxidation of olefins by using an optically active Mn-salen complex. J. Electroanal. Chem..

[18] Jacobsen E.N., Zhang W., Güler M.L. (1991). Electronic Tuning of Asymmetric Catalysts. J. Am. Chem. Soc..

[19] Pourbaix M. (1974). Atlas of Electrochemical Equilibria in Aqueous Solutions.

[20] Hincapie B., Llano S.M., Garces H.F., Espinal D., Suib S.L., Garces L.J. (2018). Epoxidation of cyclopentene by a low cost and environmentally friendly bicarbonate/peroxide/manganese system. Adsorpt. Sci. Technol..

[21] Garcia A.M., Moreno V., Delgado S.X., Ramírez A.E., Vargas L.A., Vicente M.Á., Gil A., Galeano L.A. (2016). Encapsulation of SALEN- and SALHD-Mn(III) complexes in an Al-pillared clay for bicarbonate-assisted catalytic epoxidation of cyclohexene. J. Mol. Catal. A Chem..

[22] Monfared H.H., Aghapoor V., Ghorbanloo M., Mayer P. (2010). Highly selective olefin epoxidation with the bicarbonate activation of hydrogen peroxide in the presence of manganese(III) meso-tetraphenylporphyrin complex: Optimization of effective parameters using the Taguchi method. Appl. Catal. A-Gen..

[23] Yao H., Richardson D.E. (2000). Epoxidation of alkenes with bicarbonate-activated hydrogen peroxide. J. Am. Chem. Soc..

[24] Lane B.S., Vogt M., DeRose V.J., Burgess K. (2002). Manganese-catalyzed epoxidations of alkenes in bicarbonate solutions. J. Am. Chem. Soc..

[25] Drozd V.A., Ottenbacher R.V., Bryliakov K.P. (2022). Asymmetric Epoxidation of Olefins with Sodium Percarbonate Catalyzed by Bis-amino-bis-pyridine Manganese Complexes. Molecules.

[26] Escande V., Petit E., Garoux L., Boulanger C., Grison C. (2015). Switchable Alkene Epoxidation/Oxidative Cleavage with H_2_O_2_/NaHCO_3_: Efficient Heterogeneous Catalysis Derived from Biosourced Eco-Mn. ACS Sustain. Chem. Eng..

[27] Hashimoto K., Nagatomo S., Fujinami S., Furutachi H., Ogo S., Suzuki M., Uehara A., Maeda Y., Watanabe Y., Kitagawa T. (2002). A New Mononuclear Iron(III) Complex Containing a Peroxocarbonate Ligand. Angew. Chem. Int. Ed..

[28] Richardson D.E., Yao H., Frank K.M., Bennett D.A. (2000). Equilibria, kinetics, and mechanism in the bicarbonate activation of hydrogen peroxide: Oxidation of sulfides by peroxymonocarbonate. J. Am. Chem. Soc..

[29] Zhang J., Oloman C.W. (2005). Electro-oxidation of carbonate in aqueous solution on a platinum rotating ring disk electrode. J. Appl. Electrochem..

[30] Manoharan G., Muthu Mohamed M., Raghavendran N.S., Narasimham K.C. (2000). Electrolytic preparation of sodium and potassium percarbonate. Trans. SAEST (Soc. Adv. Electrochem. Sci. Technol.).

[31] Wiel P., Janssen L.J.J., Hoogland J.G. (1971). Electrolysis of a carbonate-borate solution with a platinum anode-I. Current efficiency at perborate concentration of zero. Electrochim. Acta.

[32] Irkham, Fiorani A., Valenti G., Kamoshida N., Paolucci F., Einaga Y. (2020). Electrogenerated Chemiluminescence by in Situ Production of Coreactant Hydrogen Peroxide in Carbonate Aqueous Solution at a Boron-Doped Diamond Electrode. J. Am. Chem. Soc..

[33] Chardon C.P., Matthée T., Neuber R., Fryda M., Comninellis C. (2017). Efficient Electrochemical Production of Peroxodicarbonate Applying DIACHEM® Diamond Electrodes. ChemistrySelect.

[34] Ruiz-Ruiz E.J., Meas Y., Ortega-Borges R., Jurado Baizabal J.L. (2014). Electrochemical production of peroxocarbonate at room temperature using conductive diamond anodes. Surf. Eng. Appl. Electrochem..

[35] Velazquez-Peña S., Sáez C., Cañizares P., Linares-Hernández I., Martínez-Miranda V., Barrera-Díaz C., Rodrigo M.A. (2013). Production of oxidants via electrolysis of carbonate solutions with conductive-diamond anodes. Chem. Eng. J..

[36] Ruiz E.J., Ortega-Borges R., Jurado J.L., Chapman T.W., Meas Y. (2008). Simultaneous anodic and cathodic production of Sodium percarbonate in aqueous solution. Electrochem. Solid-State Lett..

[37] Saha M.S., Furuta T., Nishiki Y. (2004). Conversion of carbon dioxide to peroxycarbonate at boron-doped diamond electrode. Electrochem. Commun..

[38] Saha M.S., Furuta T., Nishiki Y. (2003). Electrochemical synthesis of sodium peroxycarbonate at boron-doped diamond electrodes. Electrochem. Solid-State Lett..

[39] Mavrikis S., Göltz M., Rosiwal S., Wang L., Ponce de León C. (2020). Boron-Doped Diamond Electrocatalyst for Enhanced Anodic H_2_O_2_ Production. ACS Appl. Energy Mater..

[40] Einaga Y. (2018). Development of Electrochemical Applications of Boron-Doped Diamond Electrodes. Bull. Chem. Soc. Jpn..

[41] Wenderich K., Nieuweweme B.A.M., Mul G., Mei B.T. (2021). Selective Electrochemical Oxidation of H_2_O to H_2_O_2_ Using Boron-Doped Diamond: An Experimental and Techno-Economic Evaluation. ACS Sustain. Chem. Eng..

[42] Ghosh M., Shinde V.S., Rueping M. (2019). A review of asymmetric synthetic organic electrochemistry and electrocatalysis: Concepts, applications, recent developments and future directions. Beilstein J. Org. Chem..

[43] Ma C., Fang P., Liu Z.-R., Xu S.-S., Xu K., Cheng X., Lei A., Xu H.-C., Zeng C., Mei T.-S. (2021). Recent advances in organic electrosynthesis employing transition metal complexes as electrocatalysts. Sci. Bull..

[44] Novaes L.F.T., Liu J., Shen Y., Lu L., Meinhardt J.M., Lin S. (2021). Electrocatalysis as an enabling technology for organic synthesis. Chem. Soc. Rev..

[45] Kaeffer N., Leitner W. (2022). Electrocatalysis with Molecular Transition-Metal Complexes for Reductive Organic Synthesis. JACS Au.

[46] Mavrikis S., Perry S.C., Leung P.K., Wang L., Ponce de León C. (2021). Recent Advances in Electrochemical Water Oxidation to Produce Hydrogen Peroxide: A Mechanistic Perspective. ACS Sustain. Chem. Eng..

[47] Sainna M.A., Kumar S., Kumar D., Fornarini S., Crestoni M.E., de Visser S.P. (2015). A comprehensive test set of epoxidation rate constants for iron(iv)–oxo porphyrin cation radical complexes. Chem. Sci..

[48] Bakhmutova-Albert E.V., Yao H., Denevan D.E., Richardson D.E. (2010). Kinetics and Mechanism of Peroxymonocarbonate Formation. Inorg. Chem..

[49] Zhang W., Jacobsen E.N. (1991). Asymmetric olefin epoxidation with sodium hypochlorite catalyzed by easily prepared chiral manganese(III) salen complexes. J. Org. Chem..

[50] Jacobsen E.N., Zhang W., Muci A.R., Ecker J.R., Deng L. (1991). Highly enantioselective epoxidation catalysts derived from 1,2-diaminocyclohexane. J. Am. Chem. Soc..

[51] Lane B.S., Burgess K. (2001). A Cheap, Catalytic, Scalable, and Environmentally Benign Method for Alkene Epoxidations. J. Am. Chem. Soc..

[52] Qi B., Lou L.-L., Yu K., Bian W., Liu S. (2011). Selective epoxidation of alkenes with hydrogen peroxide over efficient and recyclable manganese oxides. Catal. Commun..

[53] Dar T.A., Uprety B., Sankar M., Maurya M.R. (2019). Robust and electron deficient oxidovanadium(iv) porphyrin catalysts for selective epoxidation and oxidative bromination reactions in aqueous media. Green Chem..

[54] Kurahashi T., Fujii H. (2011). One-electron oxidation of electronically diverse manganese(III) and nickel(II) salen complexes: Transition from localized to delocalized mixed-valence ligand radicals. J. Am. Chem. Soc..

[55] McGarrigle E.M., Gilheany D.G. (2005). Chromium-and manganese-salen promoted epoxidation of alkenes. Chem. Rev..

